# Replacement of SARS-CoV-2 strains with variants carrying N501Y and L452R mutations in Japan: an epidemiological surveillance assessment

**DOI:** 10.5365/wpsar.2022.13.3.943

**Published:** 2022-09-16

**Authors:** Yusuke Kobayashi, Takeshi Arashiro, Miyako Otsuka, Yuuki Tsuchihashi, Takuri Takahashi, Yuzo Arima, Yura K. Ko, Kanako Otani, Masato Yamauchi, Taro Kamigaki, Tomoko Morita-Ishihara, Hiromizu Takahashi, Sana Uchikoba, Michitsugu Shimatani, Nozomi Takeshita, Motoi Suzuki, Makoto Ohnishi

**Affiliations:** a Center for Surveillance, Immunization and Epidemiologic Research, National Institute of Infectious Diseases, Tokyo, Japan.; b Center for Research Planning and Coordination, National Institute of Infectious Diseases, Tokyo, Japan.; c Department of Bacteriology I, National Institute of Infectious Diseases, Tokyo, Japan.

## Abstract

**Objective:**

Monitoring the prevalence of severe acute respiratory syndrome coronavirus 2 (SARS-CoV-2) variants is important due to concerns regarding infectivity, transmissibility, immune evasion and disease severity. We evaluated the temporal and regional replacement of previous SARS-CoV-2 variants by the emergent strains, Alpha and Delta.

**Methods:**

We obtained the results of polymerase chain reaction screening tests for variants conducted in multiple commercial laboratories. Assuming that all previous strains would be replaced by one variant, the new variant detection rate was estimated by fitting a logistic growth model. We estimated the transmission advantage of each new variant over the pre-existing virus strains.

**Results:**

The variant with the N501Y mutation was first identified in the Kinki region in early February 2021, and by early May, it had replaced more than 90% of the previous strains. The variant with the L452R mutation was first detected in the Kanto-Koshin region in mid-May, and by early August, it comprised more than 90% of the circulating strains. Compared with pre-existing strains, the variant with the N501Y mutation showed transmission advantages of 48.2% and 40.3% in the Kanto-Koshin and Kinki regions, respectively, while the variant with the L452R mutation showed transmission advantages of 60.1% and 71.9%, respectively.

**Discussion:**

In Japan, Alpha and Delta variants displayed regional differences in the replacement timing and their relative transmission advantages. Our method is efficient in monitoring and estimating changes in the proportion of variant strains in a timely manner in each region.

Severe acute respiratory syndrome coronavirus 2 (SARS-CoV-2) causes the coronavirus disease (COVID-19), which has rapidly spread worldwide. Novel variants have been reported, particularly with mutations in the receptor-binding domain (RBD) of the spike protein that may affect infectivity, transmissibility, immune evasion and disease severity. Based on virological characteristics and epidemic status, the World Health Organization (WHO) and other agencies have designated variants of concern (VOC), variants of interest and variants under monitoring. (*1*, *2*) By 18 October 2021, the Pango lineage B.1.1.7 and B.1.167.2 (WHO label: Alpha and Delta, respectively) were designated as VOC. B.1.1.7 and B.1.617.2 are characterized by N501Y, D614G and P168H mutations in the RBD and L452R, T487K, D614G and P618R mutations, respectively.

The Alpha variant, first identified in the United Kingdom of Great Britain and Northern Ireland in November 2020, spread rapidly nationally and then globally and was more infectious and transmissible than the earlier strains. (*3*-*5*) In Japan, the Alpha variant was first detected at the end of December 2020 among travellers from the United Kingdom and, in January 2021, in a COVID-19 patient without a history of international travel. The Delta variant demonstrated immune evasion with higher infectivity and transmissibility than previous strains. (*6*, *7*) In Japan, the Delta variant was first identified in April 2021 in a patient without a travel history, and multiple cases were detected earlier in quarantined international travellers.

Whole-genome sequencing (WGS) is used to classify SARS-CoV-2 variants, with WHO developing guidance on surveillance methods using WGS for COVID-19. (*8*) In Japan, WGS is mainly performed at the National Institute of Infectious Diseases (NIID), as well as at some local public health institutes (PHIs), university laboratories and commercial laboratories. However, testing all COVID-19 specimens by WGS has been challenging; on 27 September 2021, WGS had been conducted for  88 355 specimens, which corresponded to 5.2% of the 1 707 848 reported cases, including duplicate cases. (*9*, *10*)

Therefore, in Japan, screening tests for variants are also conducted using the polymerase chain reaction (PCR) methods developed by the NIID and commercial laboratories to detect the N501Y and L452R mutations in the Alpha and Delta variants, respectively. Since the end of March 2021, approximately 40% of patients who tested positive for COVID-19 at PHIs and commercial laboratories have undergone these PCR variant screening tests. WGS is then performed preferentially on those specimens that are positive for each mutation by the PCR variant screening test, which biases the WGS results towards strains with these mutations. (*11*)

Newly emerging variant strains, as mentioned above, may have different characteristics than pre-existing strains. It is very important to know the local status of these variant strains in the region for appropriate public health and clinical response, such as duration of isolation, estimating vaccine efficacy and selecting appropriate antiviral drugs. As far as we know, this is the first regional comparative study on variant replacement in Japan. We analysed the replacement of previous strains by variants with the N501Y and L452R mutations using region-wide data obtained from PCR screening tests and estimated the transmission advantages of the Alpha and Delta variants over pre-existing strains to describe geographical distribution differences in Japan.

## Methods

We obtained the results of PCR screening tests for variants conducted in multiple commercial laboratories, which were commissioned by the NIID for an active epidemiological investigation based on the provisions of the Act on the Prevention of Infectious Diseases and Medical Care for Patients with Infectious Diseases. In 2021, data were shared weekly with the NIID from 8 March to 17 May and from 7 June to 20 September for the variant with the N501Y mutation and the variant with the L452R mutation, respectively. Data were obtained on the numbers of specimens with and without mutations, the dates of specimen submission and the prefectures of the testing institutions. The number of commercial laboratories that provided data increased over time. Six and seven laboratories shared data on the variants with the N501Y and L452R mutations, respectively.

A sensitivity analysis was undertaken to compare data between the period when all laboratories were submitting samples and the total period. For the variant with the N501Y mutation, the sensitivity analysis compared the results from the period when data were reported from all six laboratories to those from the entire study period, including weeks when not all data were available. For the variant with the L452R mutation, the sensitivity analysis compared the results from the period when data were reported from all seven laboratories to those from the entire study period.

Assuming that all previous strains would be replaced by one variant, we estimated detection rates by fitting a daily logistic growth model to the mutation detection rates for each region. The denominator was the number of specimens with information regarding the presence of mutation, excluding those that could not be analysed by screening tests. Based on the same data, we estimated the transmission advantage of each variant over the pre-existing virus strains that were available from the genomic surveillance data. (*12*, *13*) The serial interval of COVID-19 required for the calculation was set to 4.8 days. (*14*)

We conducted analyses of the Kanto-Koshin and Kinki regions, which are metropolitan areas that include major urban centres (e.g. Tokyo and Osaka) and tend to be the centres of epidemics, plus the total for Japan. Daily numbers of COVID-19 cases reported from each region were obtained from data published by the Ministry of Health, Labour and Welfare (MHLW). (*10*) All statistical analyses were performed using R software (R Foundation for Statistical Computing, Vienna, Austria). This study was conducted under the provisions of the Act on the Prevention of Infectious Diseases and Medical Care for Patients with Infectious Diseases and did not require ethical approval, as no personally identifiable information was collected.

## Results

### Variant with the N501Y mutation

During 2021, between 8 March (week 10) and 17 May (week 20), PCR screening tests detected the N501Y mutation in 37 823 specimens, which accounted for 15.3% of the 247 962 specimens that were positive for SARS-CoV-2 during the same period, including duplicated samples (**Table 1**). Relative to the number of COVID-19 cases reported, screening was the highest in the Kanto-Koshin region (23.8 per 100 reported cases) and lowest in the Hokuriku region (3.1 per 100 reported cases) (**Table 1**). The highest number of specimens was obtained in the Kanto-Koshin region (19 369, 51.2%), followed by the Kinki region (10 108, 26.7%). Although the data were reported to the NIID each week, the average duration between the time the specimens were submitted to each laboratory and their being reported to the NIID was 9.4 days (standard deviation: 1.0 days).

**Table 1:** PCR tests conducted for N501Y and L452R mutation screening and COVID-19 cases reported by region, Japan, March to September 2021

Region	N501Y			L452R		
	8 March to 17 May 2021			7 June to 20 September 2021		
	A) Number of variant screening tests performed	B) Number of COVID-19 cases reported	A) to B) ratio	A) Number of variant screening tests performed	B) Number of COVID-19 cases reported	A) to B) ratio
Hokkaido	1955	11 302	17.3	6186	20 258	30.5
Tohoku	549	10 428	5.3	1896	19 351	9.8
Kanto-Koshin	19 369	81 471	23.8	183 315	477 227	38.4
Hokuriku	130	4187	3.1	860	13 053	6.6
Tokai	2157	21 987	9.8	14 038	90 442	15.5
Kinki	10 108	80 016	12.6	29 639	165 811	17.9
Chugoku	1023	8708	11.7	3293	22 470	14.7
Shikoku	232	4006	5.8	495	8915	5.6
Kyushu	1606	20 255	7.9	8496	65 259	13.0
Okinawa	694	5602	12.4	3565	30 322	11.8
Japan	37 823	247 962	15.3	251 783	913 108	23.6

**Fig. 1:**
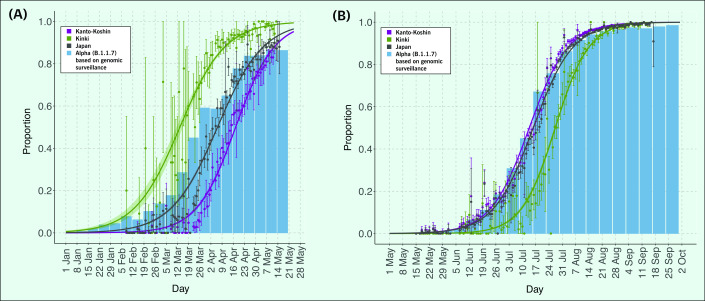
Rise in proportions of the (A) N501Y mutation and B.1.1.7 variant, January to May, and (B) L452R mutation and AY.29 (B.1.167.2) variant, May to September, Japan, 2021

In the Kinki region, the variant with the N501Y mutation was detected in early February (week 5), and by mid-April (week 15), it had replaced more than 90% of the virus strains previously circulating. In the Kanto-Koshin region, the variant with the N501Y mutation was detected in mid-February (week 6), and by mid-May (week 19), it had replaced more than 90% of the previously prevalent strains. In Japan, more than 90% of the previous virus strains were replaced by the variant with the N501Y mutation by early May (week 18; **Fig. 1A**).

[**insert** Figure 1]

**Fig. 2:**
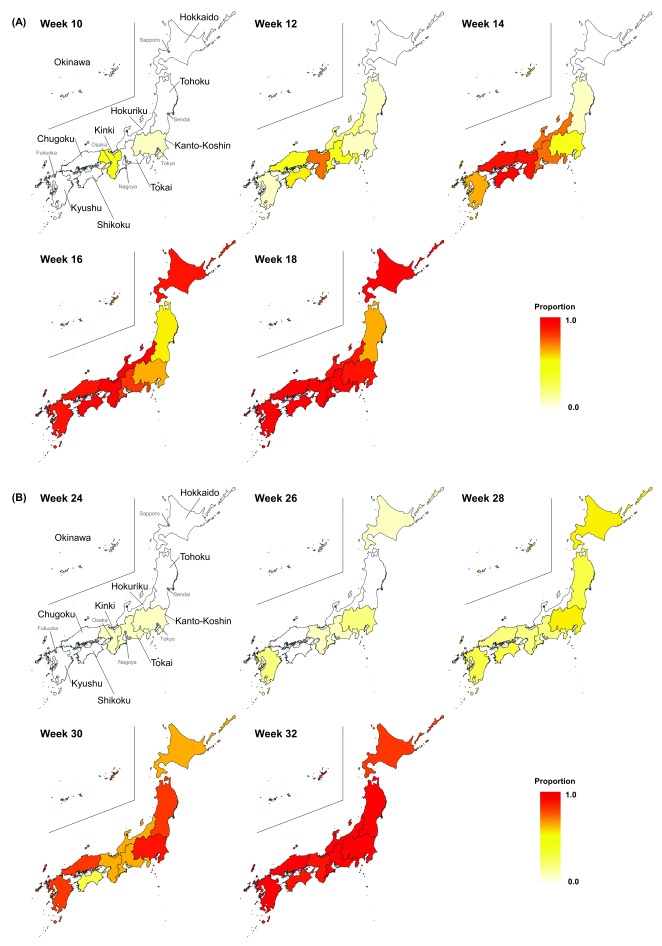
Biweekly geographical distribution of variants with the (A) N501Y (weeks 10–18) and (B) L452R (weeks 24–32) mutations by week and region, Japan, 2021

The sensitivity analysis showed that in the periods when specimens were submitted from all laboratories, compared with all study periods, 50% of the variants with the N501Y mutation were replaced by the circulated strain 4 days, 1 day and 20 days earlier in all of Japan, the Kanto-Koshin region and the Kinki region, respectively. However, 90% were replaced by the previous strain 3 and 2 days later in Japan and the Kanto-Koshin region, respectively, and 2 days earlier in the Kinki region. The proportion of samples with the N501Y mutation increased from week 10 in the Kinki region, followed by in western Japan, including the Chugoku and Shikoku regions, and in week 18, it was detected in the majority of samples in all of Japan (**Fig. 2A**).

[**insert** Figure 2]

**Fig. 3:**
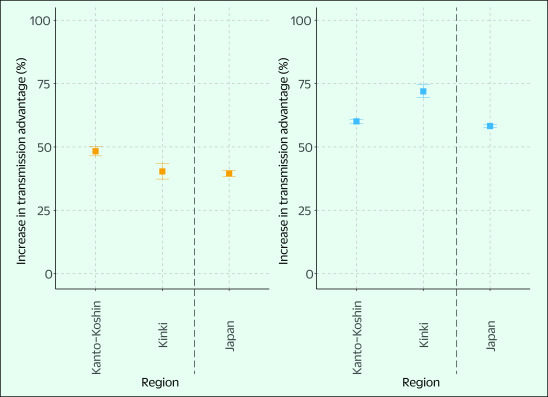
Estimated transmission advantages of the (A) N501Y mutation (March to May) and (B) L452R mutation variant (May to September) in the Kanto-Koshin region, Kinki region and Japan, 2021

The increase in transmission advantage of the variant with the N501Y mutation relative to the pre-existing virus strains was 48.2% (95% confidence interval [CI]: 46.5–50.2%) in the Kanto-Koshin region, 40.3% (95% CI: 37.3–43.5%) in the Kinki region and 39.5% (95% CI: 38.4–40.7%) nationwide (**Fig. 3**).

[**insert** Figure 3]

### Variant with the L452R mutation

Between 7 June (week 23) and 20 September (week 38), PCR screening tests to detect the variant with the L452R mutation were conducted on 251 783 specimens, which accounted for 23.6% of the 913 109 specimens reported positive for SARS-CoV-2 during the same period. Relative to the number of COVID-19 cases reported, screening was highest in the Kanto-Koshin region (38.4 per 100 reported cases) and lowest in the Shikoku region (5.6 per 100 reported cases) (**Table 1**). The highest number of specimens was obtained from the Kanto-Koshin region (183 315, 72.8%), followed by the Kinki region (29 639, 11.8%). The average interval between the time when the specimens were submitted to each laboratory and their being reported to the NIID was 8.5 days (standard deviation: 0.6 days).

In the Kanto-Koshin region, the variant with the L452R mutation was first detected in mid-May  (week 20), and by early August (week 31), it had replaced more than 90% of the virus strains previously prevalent in the region. This same variant was detected in the Kinki region in early June (week 23), and by mid-August  (week 33), it had replaced more than 90% of the previous virus strains prevalent in the region. In Japan, more than 90% of the existing virus strains had been replaced by the variant with the L452R mutation in early August (week 31) (**Fig. 1B**).

The sensitivity analysis showed that in the period when specimens were submitted from all laboratories, compared with all study periods, 50% of the variants with the L452R mutation were replaced by the circulated strain on day 0 in Japan, the Kanto-Koshin region and the Kinki region; meanwhile, 90% of the variant with the L452R mutation was replaced by the previous strain 1 day later in Japan, 1 day earlier in the Kanto-Koshin region and on day 0 in the Kinki region.

The proportion of specimens with the L452R variant in the Kanto-Koshin region increased from week 24; the Kinki and Kyushu regions followed, and at week 32, the majority of specimens in Japan were positive for this variant (**Fig. 2B**). The transmission advantage of this variant increased by 60.1% (95% CI: 59.3–60.9%) in the Kanto-Koshin region, 71.9% (95% CI: 69.4–74.5%) in the Kinki region and 58.3% (95% CI: 57.6–59.0%) nationwide, compared with the pre-existing virus strains (Alpha variant) (**Fig. 3**).

### Summary

**Fig. 4:**
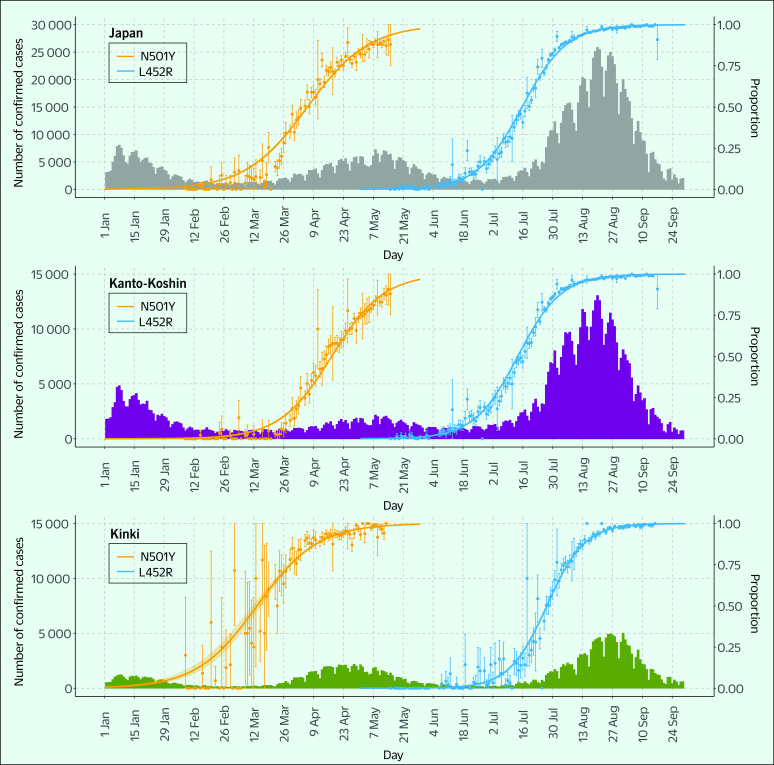
Daily counts of reported COVID-19 cases and rise in the proportions of variants with the N501Y and L452R mutations in Japan (grey), the Kanto-Koshin region (purple) and the Kinki region (green), January to September, 2021

The number of COVID-19 cases in Japan increased substantially from March to June 2021 and from July to September 2021, which coincided with increased proportions of variants with the N501Y and L452R mutations assumed to have been the dominant strain in these epidemics, respectively (**Fig. 4**).

[**insert** Figure 4]

The data and published genomic surveillance results showed that the detection rate was higher for the Alpha variant (genomic surveillance) relative to that of the variant with the N501Y mutation in screening. However, the detection rates of the Delta variant and those of the variant with the L452R mutation were almost identical (**Fig. 1**). (*11*)

## Discussion

This study revealed a rapid replacement of pre-existing virus strains by the variant with the N501Y mutation from mid-February 2021 in the Kinki region and the variant with the L452R mutation from late June in the Kanto-Koshin region, which thereafter spread throughout Japan. Relative to pre-existing virus strains, the transmission advantage of the variant with the N501Y mutation increased by 39.5% and that of the variant with the L452R mutation by 58.3%.

Various SARS-CoV-2 variants have emerged worldwide since the beginning of 2021, some of which spread rapidly and have become dominant in certain countries. In Japan, the proportion of variants with the N501Y and L452R mutations increased in line with the increased number of COVID-19 cases from March to June (fourth wave) and from July to September (fifth wave) 2021, respectively, with the increase in the proportion of these strains probably resulting in the respective epidemics. According to the WGS results, the B.1.214 strain accounted for the majority of cases in Japan from October 2020 to February 2021 (third wave), after which the number of cases with the R.1 strain increased, followed by cases with the Alpha variant. From March to June 2021 (fourth wave), the Alpha variant accounted for a large proportion of cases, while the number of cases with the R.1 strain decreased around mid-March. From late May onward, the number of cases with the B.1.617.2 variant (Delta variant; mostly reclassified as AY.29) increased, accounting for a large case proportion from July to September (fifth wave). (*11*) Therefore, it was considered that most of the cases with N501Y mutations in this study were caused by the Alpha variant, and those with L452R mutations were due to the Delta variant.

Genomic surveillance in Japan is performed on 5–10% of the specimens positive for SARS-CoV-2 and has been conducted continuously regardless of changes in the number of patients. In contrast, PCR testing to screen for a specific variant is initiated after the prediction of an epidemic caused by a particular variant strain. Therefore, genomic surveillance is more advantageous for the early detection of variant strains than PCR screening tests. Before their detection using PCR screening tests, the Alpha and Delta variants were first identified in the Kanto-Koshin region (Tokyo) in week 51 of 2020 and week 18 of 2021, respectively, based on genomic surveillance reports. As earlier genomic surveillance tended to focus on specimens testing positive by PCR screening, the proportion of specimens tested by genomic surveillance for specific mutations (i.e. N501Y and L452R mutations) was expected to be higher, resulting in a bias that overestimated the prevalence of these variant strains. However, the comparison of genomic surveillance data from this study showed no significant difference in the transition of the detection rate of either variant.

This study suggests that the proportion of cases with the N501Y mutation first increased in the Kinki region, while those with the L452R mutation initially increased in the Kanto-Koshin region. Similarly, the number of COVID-19 cases increased earlier in the Kinki region than in the Kanto-Koshin region during the fourth wave of the epidemic caused by the N501Y mutation and vice versa during the fifth wave due to the L452R mutation. Genomic surveillance reports showed that the Alpha variant was first detected in the Kanto-Koshin region. However, the subsequent rise in the percentage of cases occurred earlier in the Kinki region, which concurs with the findings of this study. The period during which Delta variant cases increased across regions corresponded to the concurrent increase in cases with L452R mutations in this study. (*11*)

The transmission advantage above pre-existing virus strains was compared for each variant. The variant with the N501Y mutation demonstrated a 39.5% rise in transmission compared to the B.1.1.214 and R1 strains, whereas that of the variant with the L452R mutation was 58.3% higher than the Alpha variant. A previous study in Japan that compared the transmission advantages of the B.1.1.7 and B.1.617.2 variants with pre-existing virus strains demonstrated an increase of 44% and 95%, respectively. (*15*) In contrast, reports from Europe and the United States of America showed that the transmission advantage of the Alpha variant increased by 42–100% compared with that of previous strains, and the Delta variant increased by 55–120% compared with that of the Alpha variant. (*13*, *16*-*19*) These values are based on the Global Initiative on Sharing Avian Influenza Data and surveillance data on the number of patients and genomic surveillance level conducted in each country, and as a result, the estimation methods might differ from those used in this study. (*20*)

Vaccination status in each country could lead to decreased transmission advantages. In Japan, COVID-19 vaccination programmes began in February 2021 for health-care workers and the older population. Increased vaccination rates might also have influenced the transmission advantage, although this could not be assessed in the present study. A retrospective survey in the Kinki region suggested that regional replacement and transmission advantage may have been due to the introduction of the Alpha variant that was not detected immediately or to a potential regional transmission that occurred earlier than detected by the investigation. (*21*) Other factors such as the composition of pre-existing strains, the timing of the introduction of each variant and the public health response to COVID-19 may have influenced the difference in transmission advantage in the Kanto-Koshin and Kinki regions. We have not been able to fully evaluate the factors that caused the differences between the regions.

This study has several limitations. First, PCR screening for variants in Japan is conducted not only in commercial laboratories but also at PHIs, some universities and hospitals. The data from commercial laboratories exhibited a regional bias in the number of specimens obtained from medical institutions. Therefore, the number of specimens might not have been sufficient to adequately evaluate the changes in the proportions of variants. In addition, there may be biases in the characteristics of the populations tested by each laboratory, such as those with a high incidence of outbreaks. Second, in the early stages of analysis, the rise in the number of laboratories might have affected the regional bias and influenced the results of regional replacement of the previous variant by that with the N501Y mutation. However, while the number of laboratories changed over time, the sensitivity analysis showed that this had little effect on the 90% replacement time. Third, in addition to the Alpha variant, the B.1.351 (Beta), P.1 (Gamma) and B.1.621 (Mu) variants have the N501Y mutation. Furthermore, with the exception of the Delta variant, B.1.617.1 (formerly Kappa) and other variants also carry the L452R mutation. When multiple variants with the same mutation are prevalent, further analysis may be required to evaluate the replacement of each mutant variant.

In conclusion, based on the PCR test results conducted at commercial laboratories to screen for the Alpha variant carrying the N501Y mutation and the Delta variant carrying the L452R mutation, we evaluated the replacement and transmissibility of the variant with the N501Y mutation and the variant with the L452R mutation compared to the B.1.1.214 and R1 strains, and Alpha strains, respectively. Our method is a reasonable and simple way to promptly monitor and estimate changes in the proportion of variant strains in each region, even in regions where genomic surveillance is not sufficiently conducted.
